# Tongue reflex for speech posture control

**DOI:** 10.1038/s41598-024-56813-9

**Published:** 2024-03-16

**Authors:** Takayuki Ito, Mohamed Bouguerra, Morgane Bourhis, Pascal Perrier

**Affiliations:** 1 Université Grenoble Alpes, CNRS, Grenoble INP (Institute of Engineering Université Grenoble Alpes), GIPSA-lab, 38000 Grenoble, France; 2 GIPSA-lab, CNRS, 11 rue des Mathématiques, Grenoble Campus BP46, 38402 Saint Martin d’Hères Cedex, France

**Keywords:** Reflexes, Motor control

## Abstract

Although there is no doubt from an empirical viewpoint that reflex mechanisms can contribute to tongue motor control in humans, there is limited neurophysiological evidence to support this idea. Previous results failing to observe any tonic stretch reflex in the tongue had reduced the likelihood of a reflex contribution in tongue motor control. The current study presents experimental evidence of a human tongue reflex in response to a sudden stretch while holding a posture for speech. The latency was relatively long (50 ms), which is possibly mediated through cortical-arc. The activation peak in a speech task was greater than in a non-speech task while background activation levels were similar in both tasks, and the peak amplitude in a speech task was not modulated by the additional task to react voluntarily to the perturbation. Computer simulations with a simplified linear mass-spring-damper model showed that the recorded muscle activation response is suited for the generation of tongue movement responses that were observed in a previous study with the appropriate timing when taking into account a possible physiological delay between reflex muscle activation and the corresponding force. Our results evidenced clearly that reflex mechanisms contribute to tongue posture stabilization for speech production.

## Introduction

The tongue is a crucial articulator in human vital functions^1^ such as swallowing, speaking and breathing. These functions can be achieved by accurately controlling tongue position over time. Tongue control is extremely robust, in particular in speech production, since humans are able to speak in an intelligible manner in extremely variable conditions, such as standing, lying, running, jumping or eating, in which the tongue undergoes a wide range of different physical constraints that vary over time. In general, such a robust control is made possible by reflex mechanisms that enable the muscles to be rapidly activated in order to counteract possible deviations from the intended movement.

However, the existence of tongue reflex in humans is still controversial. Previous studies^2,3^ failed to find any evidence for the existence of tonic stretch reflex in the human tongue, when participants were asked to maintain a tongue posture. Yet, much evidence has been found in the literature for the presence of muscle spindles^4–6^ and low-threshold mechanoreceptors^7^ in the tongue, which inform about the position and shape of the tongue and are then all potential generators of reflex signals. And indeed, in a recent behavioral study^8^, we did observe a quick compensatory movement of the tongue in response to a sudden force perturbation that pulled it in the forward direction during the production of a sustained front vowel. The compensatory reaction did not bring the tongue back to the original position, but moved it to a position that preserved the original tongue contour in the constriction region of the vocal tract. Importantly, the geometry of the constriction region determines the acoustic frequency characteristics that are relevant for a correct auditory perception of the vowel. Moreover, since this compensatory movement was induced even in an auditory masking condition^9^, it can be expected to be purely driven on a somatosensory-basis, and possibly by reflex. Although mathematical simulations using a biomechanical tongue model that incorporates stretch-like short delay feedback^8^ also support this idea, further investigations were required to clarify whether the compensatory response was driven by reflex mechanisms in response to tongue stretch.

**Figure 1:**
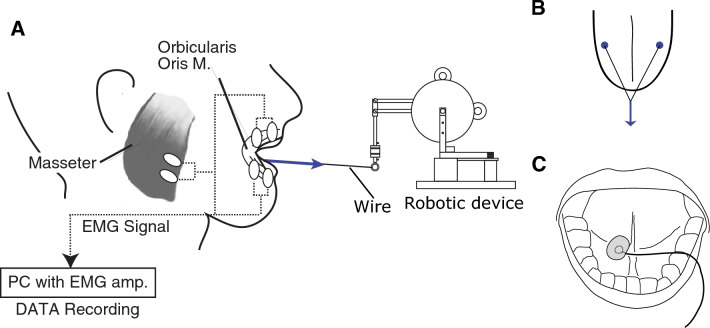
(**A**) Experimental setup with tongue perturbation and electromyographic recording. (**B**) Anchor points for the tongue perturbation. (**C**) Electrode site for the tongue EMG recording.

The current study aims to obtain physiological characterization of the tongue compensatory response for posture stabilization during vowel production. We examined the time variation of tongue muscle activations, when the force perturbation was applied to the tongue (Fig. 1A) during four different tasks: (1) the production of vowel /i/; (2) the production of the vowel /i/, with the instruction to the participant to voluntarily react to the perturbation; (3) a non-speech task, in which the participants were instructed to generate the same amount of muscle activation as in the speech task, (4) when the tongue was at rest.

For the current test, we developed an electromyographical (EMG) recording technique with a uni-polar surface electrode based on a previous study of jaw-tongue reflex^10^. The electrode was glued in the central part of the mouth floor, at the root of the tongue frenulum, in order to record activation of the anterior genioglossus, which is responsible for the control of the anterior part of the tongue^11^. A sudden stretch was applied to the tongue with a robotic device as in our previous study^8^. Although the movement of the tongue could have been measured using electromagnetic articulometer as in our previous study^8^, we did not do so, because of the difficulty to combine on the tongue surface all the sensors, electrodes and anchors used to apply the perturbation. To compensate for the absence of movement data, we carried out mathematical simulations to examine whether the observed EMG responses are compatible with the generation of the compensatory movements of the tongue that we previously observed^8^, with the appropriate timing. In this simulation the tongue was modelled as a mass-spring-damper system. In order to integrate a realistic physiological delay from EMG to muscle force, we applied the model of muscle force generation dynamics that was previously validated in an experimental study of muscle force generation mechanisms in the tongue based on electrical stimulation^12^. We evaluated how the simulated tongue displacement varied depending on the gain of the estimated force.

## Results

### EMG activation in vowel production

**Figure 2:**
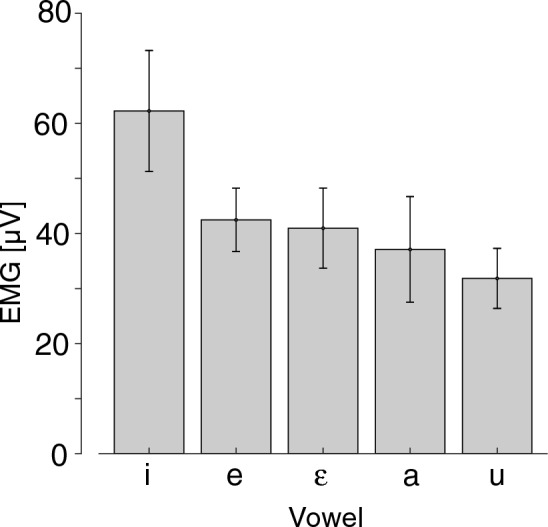
Amplitude of tongue muscle activation across vowels. Errorbars represent standard errors across the participants.

We first validated the experimental procedure by making sure that our EMG recording detects the activation of tongue muscles. Figure 2 shows the amplitude of EMG activation in each vowel. Repeated measure ANOVA shows a significant difference across vowels [F(4,28) = 4.792, p < 0.01]. The largest amplitude was found in the production of /i/, in agreement with previous observations in EMG studies of the anterior genioglossus activation using inserted electrodes^13,14^. This result supports that the recorded EMG activation reflects adequately the activation of the anterior genioglossus muscle.

### Reflex responses due to the tongue perturbation

**Figure 3:**
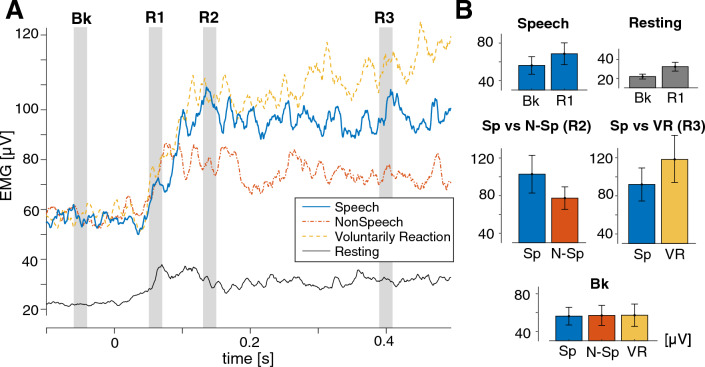
(**A**) Grand-average rectified EMG signal from the tongue muscle when the force perturbation is applied during the four different motor tasks. Time 0 corresponds to the perturbation onset. Blue solid line represents speech task. Red one-dashed line represents non-speech task. Yellow dashed line represents voluntarily reaction task. Black solid line represents resting task. Gray vertical bars represent the time windows for amplitude calculation. (**B**) Amplitudes in each comparison. *Sp* Speech task, *N-Sp* NonSpeech task, *VR* Voluntarily Reaction task. Top-left panel represents amplitudes at Bk and R1 in speech task. Top-right represents amplitudes at Bk and R1 in Resting task. Middle-left represents Sp and NonSp at R2. Middle-right represents amplitudes in Sp and VR at R3. The bottom panel represents backgound activation level (Bk) in all three tasks (Sp, N-Sp, and VR). Different colors represent each task matched with (**A**). The units for all figures are μV. Errorbars represents standard errors across the participants.

Figure 3A shows the grand-average of the rectified EMG responses measured in all the tasks. The EMG magnitude was measured at four time points: Bk, for the background activation, 50 ms before the perturbation onset; R1, for the response onset, 60 ms after perturbation onset; R2, for the activation peak, 140 ms after perturbation onset; R3, for the time after the divergence between speech and voluntarily reaction tasks (see Method for details).

### EMG response during the speech task

In Fig. 3A, the blue solid line shows the EMG signal measured during the speech task. The amplitude at time R1 was significantly larger than the background level Bk [F(1,7) = 7.73, p < 0.05] (See also the top-left panel in Fig. 3B). Paired t-tests were carried out between the EMG amplitude at time Bk and at each sample point after perturbation onset. The results revealed significant differences from 53 ms after perturbation onset. Note that we did not find any significant difference in amplitude with Bk when the temporal window was centered at 40 ms after perturbation onset [F(1,7) = 0.73, p > 0.4].

### EMG response during the resting task

The black solid-line in Fig. 3A represents the response during the resting task. The EMG amplitude increased with a similar latency as in speech task. The amplitude at time R1 was significantly larger than the background level (Bk) [F(1,7) = 6.68, p < 0.05] (see also the top-right panel in Fig. 3B). Note that a significant difference with Bk was maintained over a long time, and in particular at time R2 [F(1,7) = 6.91, p < 0.05)], and that the difference with Bk was not yet significant when the temporal window was centered at 40 ms after perturbation onset (F(1,7) = 3.046, p > 0.10). The results indicate that the observed response is not an artifact, but can be reflex. Although we did not measure significant background activation in the resting task, the reflex observed under this condition may be related to basic muscle tonus in the anterior genioglossus muscle^15^.

### EMG response due to voluntarily reaction

In the voluntarily reaction task, we verified first that vowel /i/ was similarly produced as in the speech task, by stating that there was no difference in Bk amplitude (Bk: F(1,7) = 0.04, p > 0.8). In the averaged signals, the observed reflex response was similar to the one observed in the speech task up to 200 ms after perturbation onset (yellow dashed line in Fig. 3A). Beyond this 200 ms delay, muscle activation gradually increased and diverged from the one measured in the speech task without voluntarily reaction. Rough estimation of the divergent time using paired t-tests at each sample point showed that significant difference started from 377.8 ms. Selected time-window analysis also supported that a significant difference was found at 400 ms (time point R3, Fig. 3A) (F(1,7) = 6.89, p < 0.05) (see the middle right panel in Fig. 3B). Note that no significant difference was found between these two tasks at the times R1 and R2 [R1: F(1,7) = 1.01, p > 0.3, R2: F(1,7) = 0.04, p > 0.8]. The observed latency of the voluntarily reaction, which was then longer than 375 ms, is longer than the ones formerly observed in other orofacial muscles [jaw: 150 ± 13 ms^16^, and lip: 315.7 ± 98.4 ms^17^]. This can be presumably due to a difficulty to react voluntarily while maintaining the vowel production. Overall, the comparison between the two speech tasks shows that the first part of the muscle activation induced in response to the perturbation could not to be modified voluntarily. This result provides a support for the hypothesis that the response to the perturbation was first triggered involuntarily by a reflex.

### EMG response during the non-speech task

In the non-speech task, we verified the task was executed with a similar level of muscle activation as in the speech task, by stating that there is no difference in Bk amplitude (Bk: F(1,7) = 0.05, p > 0.80). The reflex response was also induced with a similar latency (red dot-dash line in Fig. 3A). The comparison between the speech and the non-speech tasks showed a reliable difference [F(1,7) = 6.06, p < 0.05) at the peak amplitude (R2) (see the middle-left panels in Fig. 3B). These results suggest that, while reflex responses were induced in both tasks, the gain of reflex could be task-dependent. Speech task may need higher gain to more robustly preserve tongue postures.

### EMG response in the other muscles

**Figure 4:**
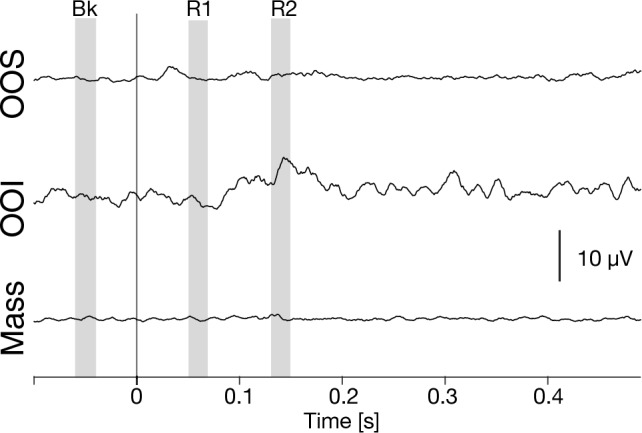
Grand-average rectified EMG signals from the three facial muscles when the force perturbation was applied during speech task. OOS is orbicularis oris superior (the upper lip muscle). OOI is orbicularis oris inferior (the lower lip muscles), and Mass is masseter (the jaw closing muscles).

There are several heterogenic reflexes from the sensory inputs in the other facial muscles, such as the jaw-tongue reflex^10^. Hence, we also considered the possibility that the sensory inputs induced in response to the tongue perturbation might induce heterogenic reflex in the facial muscles. We recorded EMG signals in lip and jaw closing muscles. Figure 4 showed the EMG responses in the upper lip muscle (OOS), lower lip muscle (OOI) and jaw closing muscle (Masseter) in speech task. Although we found some time patterns in the EMG signals that suggest the occurrence of a perioral reflex, presumably due to contacts between the thread and the lips, we found no significant variation in EMG activations of sufficient magnitude to contribute to task achievement. Heterogenic reflex appeared then not to be involved in the current context, at least functionally.

### Mathematical simulations

**Figure 5:**
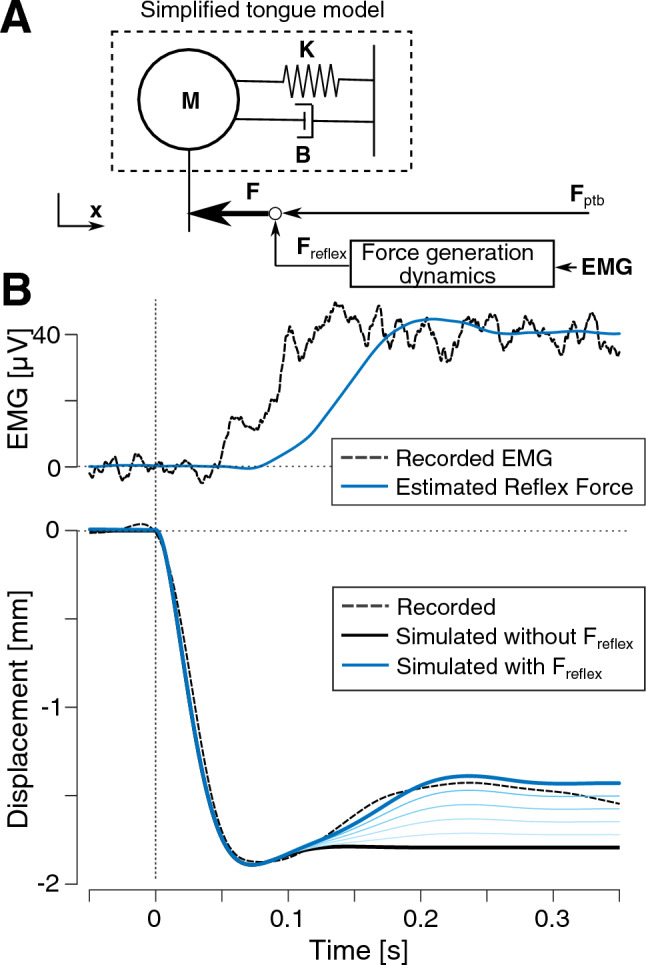
(**A**) Simplified model of tongue movement generation used in our mathematical simulation, including a model of force generation mechanisms from EMG signals (botton right) and second-order model of tongue dynamics (top left). (**B**) Simulated results. Top panel represents the recorded EMG signal and the estimated force signal. The bottom panel represents the corresponding horizontal displacements of the tongue simulated for various gain values of the reflex superimposed to the grand-average of the displacements recorded from all the participants obtained from in our previous study^8^.

Figure 5 shows the simplified tongue model with an account of force generation dynamics (Panel A) and the results of the obtained simulations (Panel B). The top panel of Fig. 5B represents the grand-average recorded EMG response (black dashed line) and the reflex force estimated using the model (blue solid lines). Because of the force generation dynamics, the temporal pattern of the reflex force is delayed and smoothed as compared to the EMG response. The bottom panel of Fig. 5B represents the grand-average displacements of the tongue recorded in Ito et al.^8^ (black dashed line) and the simulated ones using the dynamical model with various contributions of the reflex to the force: in the absence of reflex contribution (black solid line), and with increasing gains in the reflex contribution to the force (thin to thick blue solid lines); note that the thickest blue solid line corresponds to a gain magnitude that enables us to nicely fit the experimental response. We see that all the simulated displacements are identical and very close to the recorded one in the time interval [0–100 ms] after perturbation onset. However, without the inclusion of the reflex contribution to the force (black solid line), the simulated and recorded displacements diverge from each other after 120 ms. This suggests that the observed compensatory response cannot be the consequence of the only passive characteristics of tongue tissue. When we added the reflex contribution to the force with an appropriate value of the gain, the compensatory part of the recorded movement after 120 ms was nicely and qualitatively reproduced in the simulated displacement (blue thick solid line). Increasing the feedback gain made the simulated movement closer and closer to the recorded movement (thin to thick blue solid lines).

In sum, the results of our simulations support the idea that the reflex component, which is evidenced by the increase in EMG magnitude (Fig. 3A), is at the origin of the quick compensatory movement response to tongue perturbation observed in our previous study^8^.

## Discussion

In our previous study^8^, we showed a quick compensatory movement of the tongue for posture stabilization in response to a force perturbation. We hypothesized that this compensatory movement was driven by reflex mechanisms. The current study using electromyograms showed clearly an increase of tongue muscle activation (most likely with a major involvement of the anterior genioglossus) in response to the sudden tongue stretch. Importantly, the peak amplitudes (R2) of the rectified EMG signals were significantly different in the speech versus non-speech tasks and this peak was not modulated by the voluntarily reaction to the perturbation during the speech task. Mathematical simulations using the observed EMG signals reproduced the compensatory movement that was measured in our previous study, by considering a delay introduced in the account of the force generation dynamics of the tongue muscle between EMG and the generated force. This result supports our hypothesis of a crucial involvement of reflex mechanisms in the posture stabilization of the tongue, and of adaptation of the reflex gain to the specific requirements of the task.

To our knowledge, our experimental results represent the first physiological demonstration in humans that the tongue is driven by reflex in response to a sudden stretch. Previous studies failed to induce reflex in the tongue by stretching the tongue body^2,3^. In these studies, the stretch stimulation was applied manually by holding the tongue with sponge forceps or fingers. In our opinion, manual stretch has two major methodological limitations: (1) it does not ensure a consistent stimulation across trials and participants; (2) it allows the participants to anticipate the stretch and to prepare themselves resisting the force by predicting the occurrence of the force application. In contrast, the tongue perturbation used in our experiment allows for the systematic application of the external force, consistently and unpredictably, by using a robotic device. This is probably the reason why our tongue perturbation did successfully induce reflex in response to the sudden tongue stretch.

Although we did not measure displacement of the tongue simultaneously with the EMG data due to a technical complexity, our mathematical simulations with a dynamical model of muscle force generation mechanisms demonstrate that the recorded EMG data are fully compatible with the compensatory movements of the tongue that were found in response to the exact same force perturbation in our previous behavioral study^8^. Importantly the temporal pattern of the reflex force was not determined in an ad-hoc manner so as to match the recorded movement data, but was generated by considering muscle force generation dynamics that has been identified in a separate study of speech articulatory muscles including tongue muscles^12^. This model accounts for general temporal dynamics of the process between EMG signals and muscle contractions at the origin of forces. In sum, our experimental and simulation results provide a significant support for the hypothesis that the compensatory movement, which we observed in our behavioral study^3^ to stabilize the tongue posture in response to the force perturbation, has a reflex origin.

The involvement of reflex mechanisms has been already shown in studies of speech motor control using a mechanical perturbation. When the lower lip is suddenly depressed just before the intended lip closure for the production of a bilabial plosive (/p/), the activation of the upper lip muscle (OOS) increases to produce an compensatory response^18,19^, which results in a larger lowering movement of the upper lip. Similarly, a reflex was induced in the upper lip muscle when the jaw was suddenly lowered during the production of the bilabial fricative consonant /Φ/^17,20^. The gain of this reflex has can be task-specific^19,21^. The reflex compensation of the upper lip due to the lower lip perturbation was induced during bilabial explosive consonant /p/, but not during the labio-dental consonant /f/^19^. Similarly, the reflex compensation due to jaw perturbation was induced on the lip when the bilabial consonant /b/ was produced and on the tongue when the alveolar fricatives /z/ are produced^21^. The compensatory response that we observed in the tongue is in line with these findings, since the displacements of the tongue had different magnitudes between the speech and the non-speech tasks, and between vowels^8^. The current EMG data also support this view by showing that the amplitude of the reflex was reduced in the non-speech task compared with the speech task. The gain of reflex can be controlled specifically to achieve precise motor control for speech production.

The EMG signal recorded in the current study is most likely largely reflecting the activation of anterior genioglossus muscle, which controls the position of the tongue blade. However, the EMG signal could also reflect activations of other muscles that control the front-back position of the whole tongue body, namely the posterior genioglossus or the geniohyoid, which both have insertions on the mental spine close to the location of the EMG electrode. Indeed, surface EMG gathers signals from all the surrounding muscles, which cannot be separated without complex sources separation procedures. The mathematical simulations of our previous study^8^, which used a biomechanical model of the tongue showed a major contribution of the posterior genioglossus and styloglossus muscles rather than of anterior genioglossus muscles. However, whereas the time course of the simulated displacement in response to the perturbation was very close to the experimental data, we stated that the direction of the movement was not similar to the experimental one: the simulated movement was purely horizontal, moving the tongue back to its original position, while the experimental one had a significant downward vertical component (for more details see our former paper^8^, Discussion section p. 2499). Since the anterior genioglossus is a major contributor to tongue blade lowering^11^, these differences support well the hypothesis of a significant contribution of the anterior genioglossus in the experimentally observed response to the tongue stretch. Further investigation is required to clarify which muscles contribute to the observed compensatory reflex and to evaluate the simulated results from this perspective.

In the limb system, stretch reflex is known to be the fastest neural feedback response for posture stabilization. The main sensory source for stretch reflex is in muscle receptors called “muscle spindles”. The shortest latency of the stretch reflex (≈ 20 ms) involves the mono-synaptic neural arc. In the facial system, mono-synaptic stretch reflex was found in the jaw masticatory muscles known as the “jaw-jerk” reflex, which latency is around 8 ms (range 6–10 ms)^22^. Since muscle spindles have been found in tongue muscles^4^ and their density is similar to the one in jaw masticatory muscles^5^, a similar latency of response can be expected, if the mono-synaptic stretch reflex exists in the human tongue. However, in our experiment we did not observe any comparable short-latency reflex, even in resting task. Although there is still a possibility that the stimulation used in our experiment may not be fast enough to elicit muscle spindles’ response, our result suggests a lack of mono-synaptic loop arising from muscle spindles. Importantly this interpretation is consistent with the previous result that electrical stimulations of the hypoglossal nerve, which is a suitable nerve for mono-synaptic arc of stretch reflex, did not induce any reflex response in tongue muscles^2^.

The latency of the EMG response to the perturbation observed in our experiment (≈ 50 ms) is comparable to the latency of the cortical compensatory reflex in lip muscles (48.25 ± 1.2 ms), which was induced in response to jaw perturbation during the production of bilabial fricative consonants^17,20^, suggesting that the observed reflex results from a cortical processing of somatosensory feedback. In case of limb control, the reflex in response to muscle stretch is induced both in the short-latency and the long-latency phases^23^. Our previous study^8^ showed that the compensatory movement response works functionally in order to reach a posture that is not to the same as the initial posture, but is equivalent to this posture from an auditory point of view: the spectral characteristics of the speech sound associated with the initial and the compensated postures are both in the range of values associated with a correct perception of vowel /i/. Given our finding that the reflex peak in speech task was higher than in non-speech task, it can be assumed that the compensatory reflex can be tuned specifically to the speech task in order to crucially preserve the characteristics that are important for the production of perceptually relevant acoustic characteristics. Such a sophisticated reflex correction mechanism may require a cortical processing in both sensory and motor areas.

The lack of observation of mono-synaptic reflex characterized by a short latency may also suggest a possible specificity of the innervation of muscle spindles of the human tongue. The innervation is not yet known in studies performed to date. A possible spindles’ innervation could go through the hypoglossal nerve that is considered in general as a motor nerve for both intrinsic and extrinsic tongue muscles including genioglossus^24^. If this route exists, it can be assumed that the structure of the hypoglossal nerve is similar to the one of the nerves in the limb system and in the jaw. This type of nerve structure could be the base of a mono-synaptic arc that connects sensory afferents to motor neurons. A histological study of the hypoglossal nerve in the cynomolgus monkey^25^ together with the neurophysiological study in Rhesus monkeys^26^ provide support to the hypoglosso-cervical route for muscle spindles’ innervation in the tongue. However, there is no experimental evidence for any mono-synaptic arc in the human tongue, including in our data as mentioned above. Hence, the likelihood of a hypoglossal nerve contribution to the reflex observed in our experiment is low. This can be supported by the facial muscles in which no myotatic and stretch reflex has been found, and in which this type of nerve structure is missing besides the jaw closing muscles. Another possible innervation could be provided by the lingual nerve, which is considered as a sensory nerve and conveys information arising from cutaneous mechanoreceptors^7^. The tongue is a muscular hydrostat which shape and position are determined by muscles that are essentially parts of the tongue body itself. This may suggest that information about the shape of the tongue body, rather than about the length of the muscle fibres, may be used for motor control. For this purpose, cutaneous mechanoreceptors may be the main source of information instead of or together with muscle spindles. A reflex arc involving the lingual nerve has been found in the human tongue in experimental studies using electrical stimulation to the lingual nerve^27^ and percutaneous indentation on the tongue surface^28^. Despite these facts, the kinaesthetic contribution of the lingual nerve is rather controversial. The anaesthesia of the lingual nerve modified the produced speech sound suggesting a motor influence on movement^29^, whereas it rarely affects the decision when participants are asked to determine the direction of any passive movement of the tongue^30^. Although the results of the current study may support a possibility of lingual nerve innervation, further investigation is required.

Reflexes in the tongue found in previous studies ^2,27,28,31^ are probably brainstem reflex, since those latencies are shorter than 40 ms, longer that 20 ms though. Since we did not find any evidence of reflex latency shorter than 50 ms in the current test, brainstem reflexes do not seem to contribute functionally to tongue posture stabilization. Specifically, in the previous studies mentioned above, short latency reflexes were not consistently observed in all participants. Only two-third of the participants showed reflex in response to electrical stimulation (8 of 12) ^27^ and to percutaneous indentation of the tongue dorsum (6 of 10) ^28^. This may suggest that these brainstem reflexes may not work functionally in the considered motor tasks and may be suppressed depending on the task.

In the case of a mechanical perturbation, motion artifact, i.e. an EMG signal induced by the movement of the electrode under the effect of the mechanical load, can also be considered. However, this is not the case in our dataset. Indeed, if motion artifact had been the main cause of the observed variation of the EMG signal, similar variations should have been induced consistently across all perturbed trials, regardless of the condition under which the task was achieved. As a matter of fact, we tested the postural control task under various conditions in the same recording session, and we did observe different response magnitudes at the same latency in the resting task as compared to the other tasks. Since the difference is nicely related to the background EMG level, the obtained response seems to reflect the change of muscle activation in response to the tongue perturbation, but not any motion artifact.

As discussed above, various sensory organs (muscle spindles or mechanoreceptors) and nerve innervation (hypoglossal or lingual nerve) could be the source for the current reflex and this deserves further investigations. Nevertheless, the current results demonstrate the existence of an autogenic reflex response that has never been demonstrated so far in the human tongue, and they showed the functional contribution of the observed reflex to tongue postural control. The current finding can be a basis for further investigations to address which sensory receptor plays the main role to provide kinaesthetic information, and which nerve conveys in the tongue such a sensory information.

## Methods

### Participants

Eight native speakers of French participated in the experiment. The participants were all healthy young adults who reported no neurological disorders. All participants signed informed consent forms approved by the Local Ethical Committee of the University Grenoble Alpes (CERGA: Comité d’Ethique pour la Recherche, Grenoble Alpes: CERGA-Avis-2021-18). All experiments were performed in accordance with the relevant guidelines and regulations.

### Tongue perturbation

As done in our previous study^8^, a small robotic device (Phantom Premium 1.0, Geomagic) was used to apply a force to the tongue (see Fig. 1A). The robot was set in front of the participant and connected to the tongue surface through a thin thread. At both extremities of the thread, two small anchors were glued on the tongue blade surface, 2–3 cm from the tongue tip, symmetrically on both sides of the mid-sagittal plane (Fig. 1B). The tongue perturbation consisted in pulling the tongue forward with a 1-N force for 1 s. The force was applied with 5-ms rising and falling phases to avoid mechanical instabilities of the robot. The participants were required to maintain a constant stable position of the body and head.

### Electromyography (EMG)

We recorded muscle activation in the tongue from the mouth floor, anteriorly, at the root of the tongue frenulum (Fig. 1C), by using a unipolar surface electrode (Ag–AgCl). We chose unipolar recording due to the limited space on the mouth floor. This site was chosen because it is the only easily accessible site for the tongue EMG using surface electrode. Previous study^10^ demonstrated that intraoral surface recording in this selected site showed activation similar to that recorded by intramuscular electrodes in the anterior part of the genioglossus muscle, which contributes to the lowering and retraction of the tongue in its anterior part. To see potential effects of the tongue perturbation to other orofacial muscles, we also recorded EMG from the upper and lower parts of the Orbicularis Oris (OOS and OOI), which control lip aperture and protrusion, and from the masseter (MASS), which is a jaw closer. We used bipolar surface electrodes (Ag–AgCl) at the right side of the face (see the place of electrodes in Fig. 1B).

The EMG signal was amplified and filtered (band-pass: 20 Hz–5 kHz) with a biomedical amplifier (BIOPAC System, France), and sampled at 20 kHz. The recorded signal was down-sampled at 4 kHz for the analysis. During the experiment, a scrolling temporal waveform of the rectified and smoothed EMG signal from the tongue was presented on a monitor for verification and adjustment by the participants of the tongue muscle activation level as explained below.

### Experimental procedure

For the speech task, we focused on the production of the French vowel /i/. This vowel was chosen among the vowels in our previous study^8^, in which a clear compensatory response for tongue posture stabilization was induced in response to the force perturbation. In addition, the vowel /i/ is known to be associated with the largest activation of the anterior genioglossus muscle among the anterior vowels^11,13,14^. The main purpose of the current test was twofold: (1) we aim to find neurophysiological support for the reflex nature of the compensatory response that we observed at a kinematic level in our previous study^8^; (2) we aim to further investigate the specificity of the response for the speech task, as compared to a non-speech task.

In this context, in addition to the speech task, we considered three other tasks:

1. A resting task with which we verify whether the observed reflex is not due to an artifact. It is known that the amplitude of a reflex depends on the background activation levels. If the observed reflex is not an artifact, the reflex in the resting task should either be not induced or induced with a reduced amplitude.
2. A voluntarily reaction task with which we verify whether the observed response to the tongue stretch perturbation can be voluntarily modulated. A reflex is known to be automatic and be performed without voluntarily intervention. Previous studies from the literature have shown that the latency of voluntarily reactions usually exceed 200 ms^17,32^. Hence, we expected to find a confirmation of the reflex nature of the compensatory response by observing an additional muscle activation due to voluntarily reaction later than 200 ms after the perturbation onset.
3. A non-speech task with which we examine whether the observed reflex is task-specific. We expected the peak amplitude to vary depending on the task when we compared with the speech task.

The test consisted of three sessions involving different tasks in each. In all the sessions, the sequencing of each trial was as follow. The participants initiated the required task at their own pace. Then, once the rectified EMG waveform recorded from the participant’s tongue and displayed on the monitor became stable, suggesting that the postural task was achieved, the experimenter manually launched the trial. With this launching, a green circle appeared on the monitor and the participants were asked to maintain the task until the green circle disappeared, which was the signal for the end of the trial.

The first session was the main session, in which we examined whether and how muscle activation in the tongue was altered due to the tongue perturbation while participants were asked to maintain a given tongue posture. We tested three postural tasks in this session: speech, non-speech, and resting tasks. These three tasks were carried out in this order, to make easier the achievement of a level of background muscle activation which is similar in the speech and non-speech tasks, as explained below. The session consisted of three blocks. Each of the three tasks was repeated 16 times in each block, which resulted in a total of 144 trials (3 Blocks × 3 Tasks × 16 Repetitions). The mechanical perturbation was applied in randomly selected one-fourth (i.e. 12 in each task) of the trials.

In the speech task, the participants were asked to sustain the vowel /i/ for several seconds. In the non-speech task, the participants were asked to produce the same level of background muscle activation as in the speech task using any control strategy other than a speech posture control and without producing any kind of speech sound. The background muscle activation was adjusted by monitoring the rectified EMG signal. The target level of EMG amplitude was indicated on the monitor at the level that had been observed in the previous speech task trial in the absence of perturbation. The purpose of the comparison between the speech and non-speech tasks was to assess whether the reflex gain changes depending on the required task. Since reflex muscle activation is known to be proportional to the background muscle activation level, this comparison required to reach the same EMG level in both tasks. For the non-speech task, contrary to the speech task, it is difficult in general to instruct participants how to proceed or to set a specific motor task in order to reach the intended level of muscle activation. Individual variations in vocal tract and tongue morphologies also make any kind of precise recommendation useless, since feeling tongue muscle contraction and its consequence on tongue shape is typically participant dependent, and thereby cannot be shared across individuals. Hence, we asked each participant to find out by her/himself the strategy suited to achieve the target EMG amplitude with the only requirement not to use any kind of speech related motor strategy (actual and imagined) and not to push on any maxillary part such as the palate and upper teeth. As a result, the executed non-speech task might be variable across participants. This was practiced before starting the main recording and the participants found their way quickly. In the resting task, the participants were asked to be relaxed with a neutral position of the tongue. To make sure that tongue was actually resting, participants and experimenters verified on the monitor that the level of EMG activity was clearly smaller than in all the other tasks.

In the second session, we carried out the voluntarily reaction task, in order to validate that the correction mechanism observed in the first session resulted from a reflex and not from a voluntarily reaction. We used the same speech task as in the first session. The participants sustained the vowel /i/ in each trial, and they were asked to increase muscle activation as soon as they consciously perceived the perturbation force. 48 trials were carried out. The perturbation was applied in randomly selected one-fourth of trials (12 trials).

The third session aimed to verify whether the amplitude of muscle activation varies depending on the vowel, as can be assumed from former investigations^18–20^ of anterior genioglossus activations in vowel production. Since the surface electrode that we placed at the root of the tongue frenulum is supposed to largely reflect the activation of the anterior genioglossus, we can expect this amplitude to follow the experimentally found variations across vowels. We compared the amplitude of muscle activation underlying the production of the following five vowels: /i/, /e/, /ε/, /a/, and /u/. Participants were instructed via a text displayed on the monitor at the beginning of each trial. We recorded 5 repetitions of each vowel (25 trials in total).

### Data analysis

All recorded EMG data were rectified and smoothed by taking moving average using 10-ms time windows. For the data in the first and second sessions, we evaluated the effect of the task on the response to the perturbation. We analyzed the perturbed trials alone. The data were temporally aligned at the onset of the perturbation, were averaged across trials in each participant and in each task, and then the grand-average across participants was taken.

EMG amplitudes were captured using 20-ms time windows located at selected time points. The length of this time window was selected based on previous findings on the jaw-jerk reflex, which duration is more than 10 ms, and on perioral reflex, which duration is more than 15 ms on the smoothed and rectified EMG signal. To set this time window in our data set, we based on the previous findings of reflex response and voluntarily reaction task. For the onset of the reflex, we focused on several time intervals by considering different values for the reflex latency, namely [8–15] ms for the jaw-jerk reflex (an example of stretch reflex)^22^, [15–25] ms for the perioral reflex (an example of brainstem reflex)^28^ and after 50 ms for the lip compensatory reflex (an example of cortical reflex)^20^. In case of a long-latency reflex (cortical reflex), the peak of activation can be at around 90 ms or even after^17,20^. Using this knowledge and the observation of the grand-average EMG signal of the tongue muscle, we considered 20-ms time windows centered on 50 ms before perturbation onset for the background level of muscle activation (Bk), on 60 ms after perturbation onset for the onset of the compensatory response (R1), and on 140 ms after perturbation onset for the peak amplitude of the response (R2). Bk, R1 and R2 are shown with the vertical gray bars on Fig. 3A. Each time-window was used in each specific comparison.

We first examined whether the EMG of the tongue increased after the perturbation onset in the speech task. We compared the amplitudes of Bk and R1. For a rough estimation of the latency, we also applied individual paired t-test at each time point from the onset of the perturbation to the onset of the cluster showing significant difference. A similar approach was applied in the resting task. To verify whether the observed reflex was a voluntarily reaction, we compared the responses of the speech and voluntarily tasks by taking into account an additional time window (R3) to examine whether the voluntarily reaction task is associated with an increase in muscle activity. To determine R3, we carried out paired t-test between the two tasks at each sample point after the perturbation onset and detected at what time these two signals diverged. Based on this result, we centered the time window at 400 ms after the perturbation onset (see Fig. 3A). To verify whether the reflex was modulated by the task context, we compared the peak amplitude (R2) between the speech and non-speech tasks. We also compared the background EMG level between tasks to make sure that differences between tasks were not related to different initial levels of muscle activation. All the comparisons were carried out using repeated-measure ANOVA separately.

As explained above, to verify whether the recorded response is related to the muscle activation of anterior genioglossus muscle, we examined whether the amplitude of measured EMG activation during vowel production changed depending on the vowel produced. The data were collected in the third session of the test. To capture the amplitude of muscle activation for vowel production, we computed the average of EMG signal over a 20-ms time window located on the time interval where the muscle activation was stable. The obtained amplitudes were averaged across trials in each task and in each participant. Repeated-measure ANOVA was also applied across the vowels.

### Mathematical simulations

To verify whether the observed reflex EMG response can be considered to be at the origin of the compensatory response observed in tongue movement in our previous study^8^, we carried out a mathematical simulation work. In this work, we focused on the latency of the compensatory movement response. If the muscle activation measured in the present study reproduced a compensatory movement response with the appropriate latency, we can reasonably consider that the movement and EMG responses are causally linked.

In agreement with former suggestions and careful evaluations in the literature^33^, a simple mass-spring-damper model (Fig. 5A) was used to account the tongue dynamics.

$${\varvec{M}}\ddot{{\varvec{x}}}+{\varvec{B}}\dot{{\varvec{x}}}+{\varvec{K}}{\varvec{x}}={\varvec{F}}$$

where ${\varvec{x}}$ is horizontal tongue displacement, which is the main direction of the perturbation force, ${\varvec{M}}$ is mass, ${\varvec{B}}$ is damping ratio, and ${\varvec{K}}$ is stiffness ratio. ${\varvec{F}}$ includes the perturbation force and the force induced to compensate for the perturbation

$${\varvec{F}}={{\varvec{F}}}_{{\varvec{p}}{\varvec{t}}{\varvec{b}}}+\boldsymbol{ }{{\varvec{F}}}_{{\varvec{r}}{\varvec{e}}{\varvec{f}}{\varvec{l}}{\varvec{e}}{\varvec{x}}}$$

where ${{\varvec{F}}}_{{\varvec{p}}{\varvec{t}}{\varvec{b}}}$ is the perturbation force with 1 N of stepwise shape, ${{\varvec{F}}}_{{\varvec{r}}{\varvec{e}}{\varvec{f}}{\varvec{l}}{\varvec{e}}{\varvec{x}}}$ is the force induced by the reflex response. Importantly, this reflex force was determined by a model of muscle force generation dynamics, ${\varvec{D}}({\varvec{s}})$, which represents the relation between EMG activation and muscle force ^34^, described in the Laplace transform domain as follows:

$${{\varvec{F}}}_{{\varvec{r}}{\varvec{e}}{\varvec{f}}{\varvec{l}}{\varvec{e}}{\varvec{x}}}={\varvec{D}}\left({\varvec{s}}\right)\boldsymbol{ }\cdot {\varvec{E}}{\varvec{M}}{\varvec{G}}$$

$${\varvec{D}}\left({\varvec{s}}\right)=\frac{{\text{G}}{{ \omega }_{n}}^{2}}{{s}^{2}+2\zeta {\omega }_{n}+ {{\omega }_{n}}^{2}}{e}^{-\tau s}$$

where $s$ is the Laplacian operator, ${\varvec{E}}{\varvec{M}}{\varvec{G}}$ is the input EMG signal, ${\omega }_{n}$ is the natural frequency, $\zeta$ is the damping ratio, $\tau$ is the time delay and ${\varvec{G}}$ is the gain.

From a physiological perspective, this force generation dynamics can be interpreted as follows: (1) the second-order dynamics represents the combination of the chemical dynamics of the variation of calcium concentration in muscle fibers and of the mechanical dynamics for sliding filament; (2) the time-delay $\tau$ represents the neural transmission delay and the chemical transmission delay of muscle contraction^12^. The coefficients corresponding to tongue muscles were estimated in a previous study^11^ as follows: ${\omega }_{n}=6.11Hz$, $\zeta =0.675$, $\tau =17.35ms$. The gain ${\varvec{G}}$ is an adjustment parameter adapted to reduce the error between the amplitudes of the recorded and simulated displacements. For the estimation of the force, the input signal was the grand-average of the rectified and smoothed EMG signal. The background level of the input signal was adjusted to zero.

We first estimated ${\varvec{M}},\boldsymbol{ }{\varvec{B}}$ and ${\varvec{K}}$ by using the tongue movement data in the horizontal direction recorded in our previous study^8^ and the perturbation force ${{\varvec{F}}}_{{\varvec{p}}{\varvec{t}}{\varvec{b}}}$ that set for the best-fit mentioned below. In order to facilitate the estimation of these parameters, we considered that the initial part of the tongue displacement is the indicial response of the tongue. This is why we approximated the perturbation force as a step function, instead as a linearly increasing pattern as in the experiment, with a 5 ms delay from the recorded onset time.

As a result of the best-fit concerning the initial part of the response, we applied the data until 80 ms after perturbation onset including 220 ms of pre-stimulus intervals. Then we carried out the simulation to reproduce the recorded displacement using the estimated parameters, ${\varvec{M}},\boldsymbol{ }{\varvec{B}}$ and ${\varvec{K}}$, and the force including the perturbation force, ${{\varvec{F}}}_{{\varvec{p}}{\varvec{t}}{\varvec{b}}}$ and the reflex force, ${{\varvec{F}}}_{{\varvec{r}}{\varvec{e}}{\varvec{f}}{\varvec{l}}{\varvec{e}}{\varvec{x}}}$. We tested several amplitudes of the gain in order to examine how the simulated displacement was changed in function of the gain.

## Data Availability

The datasets generated during and/or analyzed during the current study are available from the corresponding author on reasonable request.

## References

[1] Pennisi E (2023). Tales of the tongue. Science.

[2] Bratzlavsky M, van der Ecken H (1974). Afferent influences upon human genioglossal muscle. J. Neurol..

[3] Neilson PD, Andrews G, Guitar BE, Quinn PT (1979). Tonic stretch reflexes in lip, tongue and jaw muscles. Brain Res..

[4] Cooper S (1953). Muscle spindles in the intrinsic muscles of the human tongue. J. Physiol..

[5] Kubota K, Negishi T, Masegi T (1975). Topological distribution of muscle spindles in the human tongue and its significance in proprioception. Bull. Tokyo Med. Dent. Univ..

[6] Saigusa H, Yamashita K, Tanuma K, Saigusa M, Niimi S (2004). Morphological studies for retrusive movement of the human adult tongue. Clin. Anat..

[7] Trulsson M, Essick GK (1997). Low-threshold mechanoreceptive afferents in the human lingual nerve. J. Neurophysiol..

[8] Ito T, Szabados A, Caillet J-L, Perrier P (2020). Quick compensatory mechanisms for tongue posture stabilization during speech production. J. Neurophysiol..

[9] Bourhis, M., Perrier, P., Savariaux, C. & Ito, T. Compensatory movement of the tongue for speech production with or without masking noise. In *SMC 2022—8th International Conference on Speech Motor Control* (Groningen, Netherlands, 2022).

[10] Ishiwata Y, Hiyama S, Igarashi K, Ono T, Kuroda T (1997). Human jaw-tongue reflex as revealed by intraoral surface recording. J. Oral. Rehabil..

[11] Buchaillard S, Perrier P, Payan Y (2009). A biomechanical model of cardinal vowel production: Muscle activations and the impact of gravity on tongue positioning. J. Acoust. Soc. Am..

[12] Ito T, Murano EZ, Gomi H (2004). Fast force-generation dynamics of human articulatory muscles. J. Appl. Physiol..

[13] Miyawaki K, Hirose H, Ushijima T, Sawashima M (1975). A preliminary report on the electromyographic study of the activity of the lingual muscles. Ann. Bull. RILP.

[14] Baer T, Alfonso PJ, Honda K (1988). Electromyography of the tongue muscles during vowels in /әpVp/ environment. Annu. Bull. RILP.

[15] Valbuza JS (2010). Methods for increasing upper airway muscle tonus in treating obstructive sleep apnea: Systematic review. Sleep Breath.

[16] Ottenhoff FA, van der Bilt A, van der Glas HW, Bosman F (1992). Peripherally induced and anticipating elevator muscle activity during simulated chewing in humans. J. Neurophysiol..

[17] Ito T, Kimura T, Gomi H (2005). The motor cortex is involved in reflexive compensatory adjustment of speech articulation. Neuroreport.

[18] Gracco VL, Abbs JH (1985). Dynamic Control of perioral system during speech: Kinematic analysis of autogenic and nonautogenic sensorimotor processes. J. Neurophysiol..

[19] Shaiman S, Gracco VL (2002). Task-specific sensorimotor interactions in speech production. Exp. Brain Res..

[20] Gomi H, Ito T, Murano EZ, Honda M (2002). Compensatory articulation during bilabial fricative production by regulating muscle stiffness. J. Phon..

[21] Kelso JAS, Tuller B, Vatikiotis-Bateson E, Fowler CA (1984). Functionally specific articulatory cooperation following jaw perturbations during speech: Evidence for coordinative structures. J. Exp. Psychol. Hum. Percept. Perform..

[22] Murray GM, Klineberg IJ (1984). Electromyographic recordings of human jaw-jerk reflex characteristics evoked under standardized conditions. Arch. Oral. Biol..

[23] Pruszynski JA, Scott SH (2012). Optimal feedback control and the long-latency stretch response. Exp. Brain Res..

[24] Loh C, Maya MM, Go JL (2002). Cranial nerve XII: The hypoglossal nerve. Semin. Ultrasound CT MR.

[25] Fitzgerald MJ, Sachithanandan SR (1979). The structure and source of lingual proprioceptors in the monkey. J. Anat..

[26] Bowman JP, Combs CM (1968). Discharge patterns of lingual spindle afferent fibers in the hypoglossal nerve of the rhesus monkey. Exp. Neurol..

[27] Maisonobe T (1998). Reflexes elicited from cutaneous and mucosal trigeminal afferents in normal human subjects. Brain Res..

[28] Weber CM, Smith A (1987). Reflex responses in human jaw, lip, and tongue muscles elicited by mechanical stimulation. J. Speech Hear. Res..

[29] Niemi M (2002). Effects of transitory lingual nerve impairment on speech: An acoustic study of vowel sounds. J. Oral Maxillofac. Surg..

[30] Adatia AK, Gehring EN (1971). Proprioceptive innervation of the tongue. J. Anat..

[31] Lowe AA, Gurza SC, Sessle BJ (1977). Regulation of genioglossus and masseter muscle activity in man. Arch. Oral Biol..

[32] Cole KJ, Abbs JH (1987). Kinematic and electromyographic responses to perturbation of a rapid grasp. J. Neurophysiol..

[33] Fuchs S, Perrier P, Hartinger M (2011). A critical evaluation of gestural stiffness estimations in speech production based on a linear second-order model. J. Speech Lang. Hear. Res..

[34] Mannard A, Stein RB (1973). Determination of the frequency response of isometric soleus muscle in the cat using random nerve stimulation. J. Physiol..

